# The binding orientation of epibatidine at α7 nACh receptors

**DOI:** 10.1016/j.neuropharm.2017.01.008

**Published:** 2017-04

**Authors:** Andrew J. Thompson, Simon Metzger, Martin Lochner, Marc-David Ruepp

**Affiliations:** a Department of Chemistry and Biochemistry, University of Bern, Bern, Switzerland; b Department of Pharmacology, University of Cambridge, Cambridge, UK; c Institute of Biochemistry and Molecular Medicine, University of Bern, Bern, Switzerland

**Keywords:** Cys-loop, Ion channel, Radioligand, Epibatidine, Agonist, α7 nACh, Nicotinic, Receptor, AChBP, 5-HT, 5-hydroxytryptamine, nACh, nicotinic acetylcholine, GABA, gamma-aminobutyric acid, HEK, human embryonic kidney, AChBP, acetylcholine binding protein, 5HTBP, an AChBP mutant modified to resemble the 5-HT_3_R binding site

## Abstract

Epibatidine is an alkaloid toxin that binds with high affinity to nicotinic and muscarinic acetylcholine receptors, and has been extensively used as a research tool. To examine binding interactions at the nicotinic receptor, it has been co-crystallised with the structural homologue acetylcholine binding protein (AChBP; PDB ID 2BYQ), and with an AChBP chimaera (3SQ6) that shares 64% sequence identity with the α7 nACh receptor. However, the binding orientations revealed by AChBP co-crystal structures may not precisely represent their receptor homologues and experimental evidence is needed to verify the ligand poses. Here we identify potential binding site interactions between epibatidine and AChBP residues, and substitute equivalent positions in the α7 nACh receptor. The effects of these are probed by [^3^H]epibatidine binding following the expression α7 nACh receptor cysteine mutants in HEK 293 cells. Of the sixteen mutants created, the affinity of epibatidine was unaffected by the substitutions Q55C, L106C, L116C, T146C, D160C and S162C, reduced by C186A and C187A, increased by Q114C and S144C, and abolished by W53C, Y91C, N104C, W145C, Y184C and Y191C. These results are consistent with the predicted orientations in AChBP and suggest that epibatidine is likely to occupy a similar location at α7 nACh receptors. We speculate that steric constraints placed upon the C-5 position of the pyridine ring in 3SQ6 may account for the relatively poor affinities of epibatidine derivatives that are substituted at this position.

## Highlights

• The binding orientation of epibatidine is known from AChBP crystal structures.
• In silico docking shows this orientation is conserved across AChBP proteins.
• By mutagenesis we confirm a similar orientation in α7 nACh receptors.
• This suggests a basis for designing epibatidine-based probes.

## 1 Introduction

α7 nACh receptors are considered to be valuable drug targets as they modulate many physiological responses and are associated with neurological disorders and pathological conditions such as Alzheimer's, Parkinson's and schizophrenia (Hernandez and Dineley, 2012, Mazurov et al., 2011, Thomsen et al., 2010). Consequently there have been continued efforts to develop new ligands that target these receptors, both as drug candidates and as pharmacological research tools. Knowledge of how the ligands orientate in the receptor binding site can provide insights into where modifications might be tolerated. Such changes can be used to improve their pharmacological properties and aid the coupling of functional groups such as fluorescent labels, thiol-reactive moieties and photo-affinity probes (Lochner and Thompson, 2015).

**Fig. 1:**
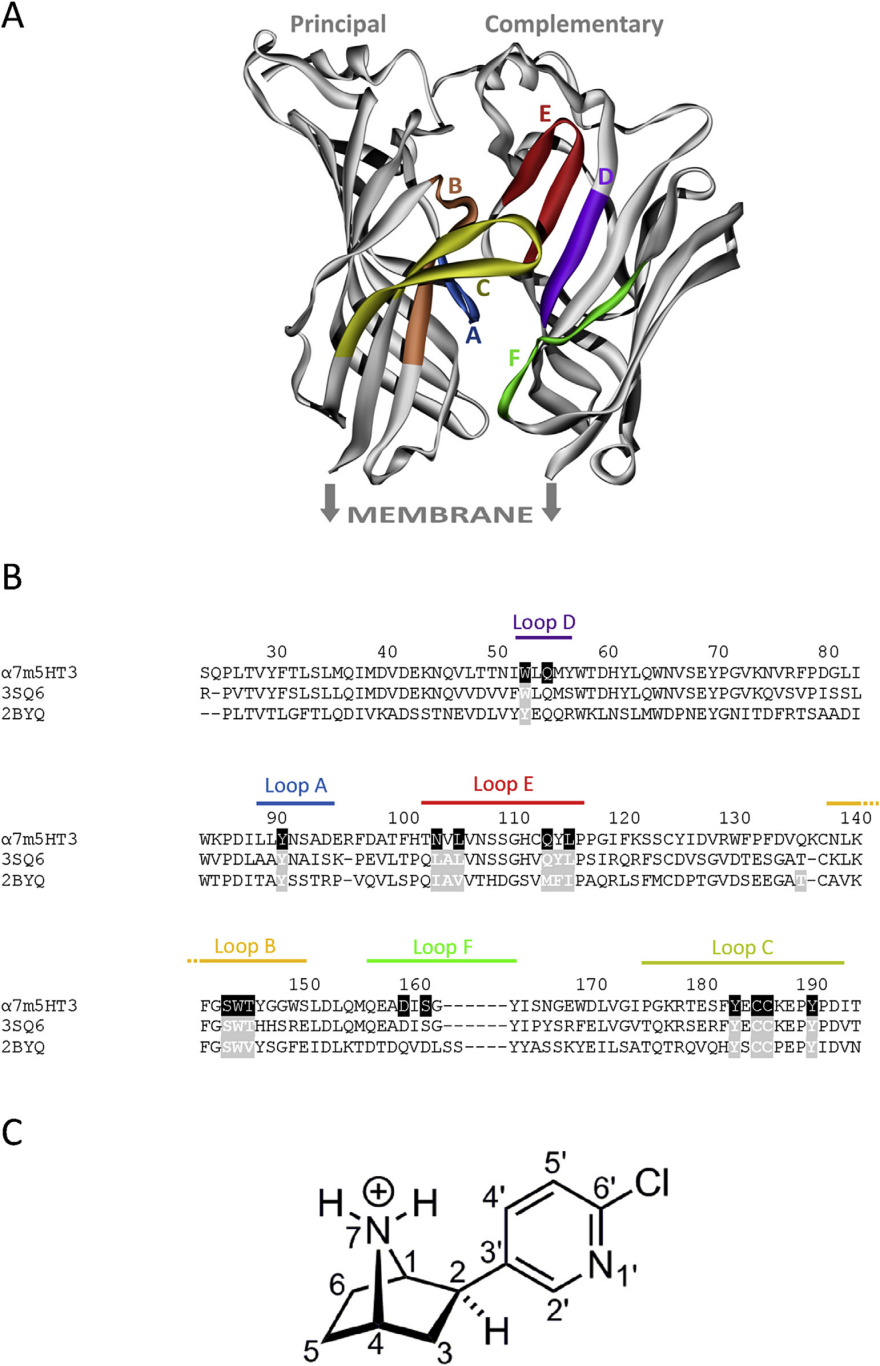
Locations of residues mutated in this study. **A**. A cartoon showing the binding site of AChBP with binding loops A – F highlighted in colour. The binding site is found at the interface of two adjacent subunits, and for clarity only two of the five subunits of the native receptor are shown. The structure is 3SQ6, but the location of the membrane has been indicated to show where it would be located in the α7 nACh receptor. **B**. An amino acid sequence alignment of the α7 nACh receptor and sequences from the two crystal structures (2BYQ & 3SQ6) in which epibatidine has been co-crystallised. The binding loops are shown in the same colours as in panel A, and amino acids that are within 5 Å of epibatidine are highlighted as white text in grey boxes on the AChBP structures. The equivalent α7 nACh residues substituted in this study are shown as white text in black boxes. α7m5HT3 = chick α7nACh chimaera; 3SQ6 = *Ls*-AChBP-α7 nACh chimaera; 2BYQ = *Ac*-AChBP. The residue numbering used in this manuscript corresponds to residues in the crystal structure 3SQ6. 2BYQ and 3SQ6 share 31.8% identity and 49.8% similarity in their sequences (EMBOSS Needle; Rice et al., 2000). **C**. The structure and atom numbering of protonated epibatidine is shown below the alignment.

α7 nACh receptors belong to the Cys-loop family of transmembrane ligand-gated ion-channels that are responsible for fast synaptic neurotransmission in the central and peripheral nervous systems. All members of this family are composed of five subunits, each of which contains an extracellular, a transmembrane and an intracellular domain (Thompson et al., 2010; Unwin, 2005). Competitive ligands such as acetylcholine or epibatidine bind at an extracellular, orthosteric binding site that is located at the interface of two adjacent subunits (Fig. 1). This site is a hydrophobic cavity created by the convergence of loops A – C from the principal subunit and loops D – F from the complementary subunit (Fig. 1). However, owing to the difficulty of crystallising membrane proteins, a soluble structural homologue (acetylcholine binding protein; AChBP), has been commonly used to study the binding orientations of α7 nACh receptor ligands. Examples include the agonists acetylcholine (3WIP), anabasine (2WNL), carbamylcholine (1UV6), cytisine (4BQT), epibatidine (2BYQ, 3SQ6), nicotine (1UW6), tropisetron (2WNC) and varenicline (4AFT, 4AFG), and antagonists such as lobeline (2BYS, 4AFH, 5AFH) and methyllycaconitine (2BYR). Owing to the relatively high sequence similarity of AChBP and the α7 nACh receptor (≤64%) it is generally thought that the orientations of ligands in the crystals will be similar to those in the α7 nACh receptor. Consequently these structures have been used to direct efforts in the chemical synthesis of novel ligands and for computational methods such as molecular dynamic simulations (Akdemir et al., 2012, Camacho-Bustamante et al., 2016, Yamauchi et al., 2012). While several studies report the successful creation of novel α7 nACh receptor ligands, AChBP co-crystal structures should still be viewed with caution as some of the ligands they contain also bind to other Cys-loop family members where they have different affinities and functional effects. For example, varenicline inhibits nACh receptors, but is a partial agonist of 5-HT_3_ receptors (Lummis et al., 2011). Similarly, tropisetron is a partial agonist of α7 nACh receptors and a high–affinity antagonist of 5-HT_3_ and glycine receptors (Macor et al., 2001, Yang et al., 2007). Such dramatic pharmacological differences suggest that some ligands may have distinct orientations at the different Cys-loop receptors and that the AChBP structures are not always representative of all family members.

Here we probe residues that lie within 5 Å of bound epibatidine in the AChBP crystal structures 2BYQ and 3SQ6, and residues identified by *in silico* docking into AChBP homology models. By substituting the equivalent residues at α7 nACh receptors we determine whether their effects on [^3^H]epibatidine binding are consistent with the ligand orientations predicted from the structures.

## 2 Materials and methods

### 2.1 Computational analysis

To gain insights into potential binding-site interactions, residues located within 5 Å of epibatidine were identified from two AChBP crystals structures (2BYQ & 3SQ6). Additionally, *in silico* ligand docking was performed on epibatidine docked into AChBP crystal structures bound with the α7 nACh receptor agonists acetylcholine (3WIP), anabasine (2WNL), carbamylcholine (1UV6), cytisine (4BQT), nicotine (1UW6), tropisetron (2WNC) and varenicline (4AFT). Agonist-bound structures were chosen as these are likely to have more appropriate binding site conformations than antagonist-bound structures; the conformational changes induced by agonist binding can have dramatic effects on ligand binding (Colquhoun, 1998). The calculated p*K*_a_ value of the bicyclic amine of epibatidine (Fig. 1) was 10.5 using MarvinSketch v15.3 and therefore the protonated and three-dimensional structure of epibatidine was constructed ab initio using Chem3D Pro v14.0 (CambridgeSoft, Cambridge, UK). The generated ligand was subsequently energy-minimised using the implemented MM2 force field. The binding site in AChBP was defined as being within 10 Å of the centroid of the aromatic side-chain of W145, a residue that is centrally located in the binding site and known to be important for the binding of competitive ligands (Thompson et al., 2010). Epibatidine was docked using GOLD Suite v5.3 (The Cambridge Crystallographic Data Centre, Cambridge, UK) with the GoldScore function and default settings. For each AChBP template, ten docking poses were generated. All residues within 5 Å of epibatidine were identified in each of docked poses and these residues and their potential interactions visualised using PyMol v1.3 (Schröndinger, NY, USA).

### 2.2 Receptors

Mutagenesis was performed using the QuikChange method (Agilent Technologies Inc., CA, USA) to substitute amino acids throughout each of the binding loops A – F (Fig. 1). The α7 nACh-5-HT3 subunit cDNA was as described by Bertrand (Eiselé et al., 1993) and was cloned into pcDNA3.1 for expression in HEK293 cells. Human 5-HT3A cDNA (accession number: P46098) and mouse 5-HT3A cDNA (Q6J1J7) were cloned into the same vector. Cells were transiently transfected with this cDNA using either polyethyleneimine (PEI: 25 kDa, linear, powder, Polysciences Inc., Eppelheim, Germany) or Lipofectamine 2000 (Life Technologies, CA, USA) according to the manufacturer's instructions. 30 μl of PEI (1 mg ml^−1^), 5 μg cDNA and 1 ml DMEM/F12 were incubated for 10 min at room temperature, added drop wise to a 90 mm plate of 70–80% confluent HEK293 cells, and incubated for 2–3 days before use.

### 2.3 Cell culture and transfection

Human embryonic kidney (HEK) 293 cells were grown on 90 mm round tissue culture plates as monolayers in DMEM/F12 (Life Technologies) supplemented with 10% fetal bovine serum (FBS; Sigma Aldrich) at 37 °C in a moist atmosphere containing 5% CO_2_.

### 2.4 Radioligand binding

Transfected HEK 293 cells were scraped into 0.6 ml of ice-cold 10 mM HEPES buffer (pH 7.4) and stored frozen. After thawing, they were washed with HEPES buffer, homogenised by passage through a 21 G needle, and 50 μg of cell suspension incubated in 0.5 ml HEPES buffer containing [^3^H]epibatidine (55.8 Ci/mmol, Waltham, MA). Non-specific binding was determined using 300 μM (−)-nicotine. Equilibrium reactions were incubated on ice for at least 1 h. Incubations were terminated by vacuum filtration onto Whatman GF/B filters wetted with 0.3% polyethyleneimine, followed by two rapid washes with 2.5 ml ice cold buffer. Radioactivity was determined by scintillation in Ultima Gold XR (Perkin Elmer) using a Tri-Carb 2100 TR (PerkinElmer) scintillation counter. Final counts were monitored to ensure that binding never exceeded 10% of the added concentrations of radioligands.

### 2.5 Data analysis

All data were analysed using GraphPad Prism v5. Individual saturation binding experiments were fitted to Eq. (1):

(1) $$y=B_{\max}\times \left[L\right]\frac{K_{d}}{K_{d}+\left[L\right]}$$

where B_max_ is maximum binding at equilibrium, *K*_d_ is the equilibrium dissociation constant and [L] is the free concentration of radioligand. Values from individual experiments were averaged to yield the mean ± sem. Affinities were compared using 1-way ANOVA and a Dunnett's Post-Test.

### 2.6 Detection

48 h post transfection, HEK293 cells were harvested in 10 ml phosphate buffered saline (137 mM NaCl, 10 mM Na_2_HPO_4_, 2.7 mM KCl, 2 mM KH_2_PO_4_, pH 7.4), centrifuged 5 min at 220 g and the pellets stored at −80 °C. Total extracts were prepared by resuspension of 1 × 10^7^ cells in 1 ml lysis buffer (10 mM Tris-HCl pH 7.5, 10 mM NaCl, 2 mM EDTA, 5 mM MnSO_4_, 4× Halt Protease Inhibitor (Thermo Fisher Scientific), 0.2 mg ml^−1^ RNase A (Sigma-Aldrich), 500 U ml^−1^ Cyanase (RiboSolutions, TX, USA), 0.1% Triton X-100) followed by 30 min on ice. Extracts were supplemented with NuPage LDS buffer (Thermo Fischer Scientific) and heated at 70 °C for 10 min. 10^5^ cell equivalents were loaded on a 4–12% Bis-Tris NuPage gel and blotted on nitrocellulose using the iBlot system (ThermoFisher Scientific). α7 nACh-5-HT_3_ chimaeras were detected with 1:250 rabbit polyclonal antibody (ASR-031; Alomone Labs, Jerusalem, Isreal) and 1:10000 goat anti-rabbit IRDye800CW (926-32211; LI-COR Biosciences, NE, USA). CPSF-100 served as a loading control and was detected using 1:10000 rabbit polyclonal antibody (kindly provided by Georges Martin & Walter Keller, University of Basel) and 1:10000 goat anti-Rabbit IRDye680LT (925-68021, LI-COR Biosciences). Signals were improved using SuperSignal Western Blot Enhancer (Thermo Fischer Scientific) according to the manufacturer's instructions.

## 3 Results & discussion

[^3^H]epibatidine displayed high-affinity saturable binding at α7 nACh receptors with a *K*_d_ (8 nM) similar to that reported elsewhere (Li et al., 2011, Xiao et al., 2012). To better understand the residues that govern this binding interaction, elsewhere crystal structures of homologous AChBP have been used as substitutes for this receptor (Hansen et al., 2005, Li et al., 2011). Here we examined whether these co-crystal structures are good representations of epibatidine binding at α7 nACh receptors and used them to speculate why chemical modification of this ligand may have previously generated much lower affinity fluorescent derivatives (Grandl et al., 2007). To this end we identified potential ligand-receptor interactions by examining the orientations of epibatidine in AChBP crystal structures and following *in silico* docking into other agonist-bound AChBP structures. Using radioligand binding we determined whether these interactions are consistent with the effects of substituting equivalent residues in the α7 nACh receptor.

**Fig. 2:**
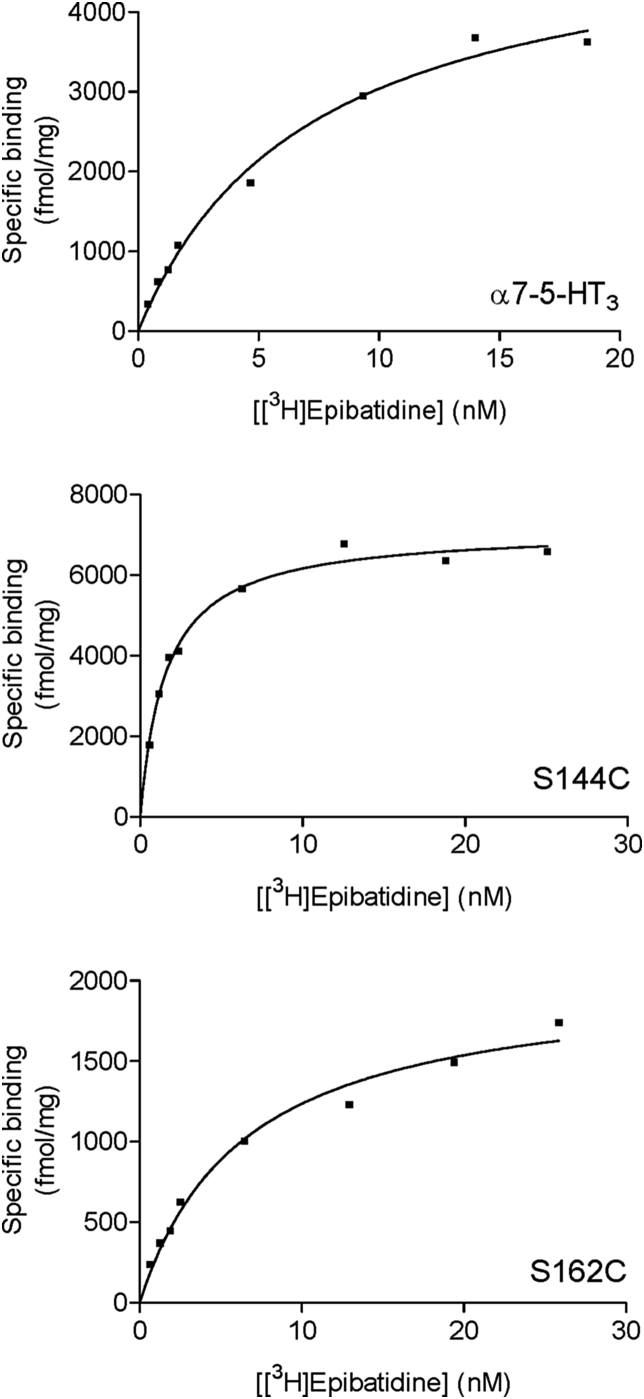
Example binding curves for [^3^H]epibatidine at wild type and mutant α7 nACh receptors.

**Fig. 3:**
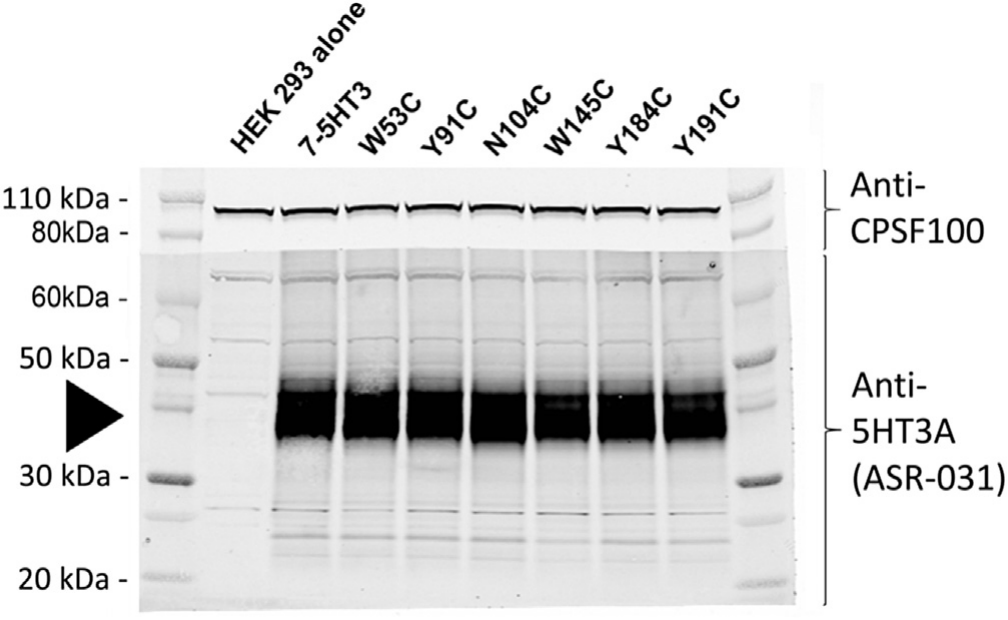
Expression of mutant α7nACh receptors. Several mutant receptors did not bind [^3^H]epibatidine. To confirm that this was a change in the binding affinity rather than altered expression, these mutants were probed by Western blot using a receptor-specific antibody directed against the 5-HT_3_ region of the chimaeric receptor. Expression levels of the mutants and wild type receptors were comparable (black arrow), and there was no detection in untransfected cells. In each lane 10^5^ cell equivalents were loaded. The membrane was cut at 70 kDa and the upper section was incubated with an antibody of cleavage and polyadenylation specificity factor (CPSF) to provide an internal control. For both Western blots and radioligand binding the same whole-cell homogenates were used, ensuring that the same population of receptors was probed.

Residues within 5 Å of epibatidine were identified in the co-crystal structures 2BYQ and 3SQ6, and following *in silico* docking into seven other agonist-bound AChBP templates; acetylcholine (3WIP), anabasine (2WNL), carbamylcholine (1UV6), cytisine (4BQT), nicotine (1UW6), tropisetron (2WNC) and varenicline (4AFT). For each template 10 docked poses were generated, yielding a total of 72 independent ligand orientations when both these and the crystal structures are considered (Table 1). However, when all poses were compared there was very little difference between orientations of epibatidine (RMSD = 0.78 Å average), suggesting that its orientation is tightly confined by the agonist-bound conformation. For each of the main residues identified in these poses, Cys substitution at the equivalent residue positions in the α7 nACh receptor was undertaken and the binding affinity of [^3^H]epibatidine at these mutants was measured (Fig. 2, Table 2). Changing 6 of the 16 residues resulted in no significant change in affinity when compared to wild type α7 nACh receptors (Q55, L106, L116, T146, D160, S162), suggesting these residues do not play a significant role in ligand binding or the conformation of the binding site. For the remaining 10 mutants there were differences in the binding affinities. Of these, 2 had increased affinities (Q114C, S144C), 2 had decreased affinities (C186A, C187A) and 6 showed no saturable binding (*K*_d_ > 20 nM; W53C, Y91C, N104C, W145C, Y184C, Y191C). For mutants that did not show saturable binding, Western blots were performed to confirm receptor expression (Fig. 3). The observed signals showed that all mutants were expressed and the absence of measurable binding could be attributed to changes in binding site interactions, or to altered gating which can also impact the affinity (Colquhoun, 1998).

**Fig. 5:**
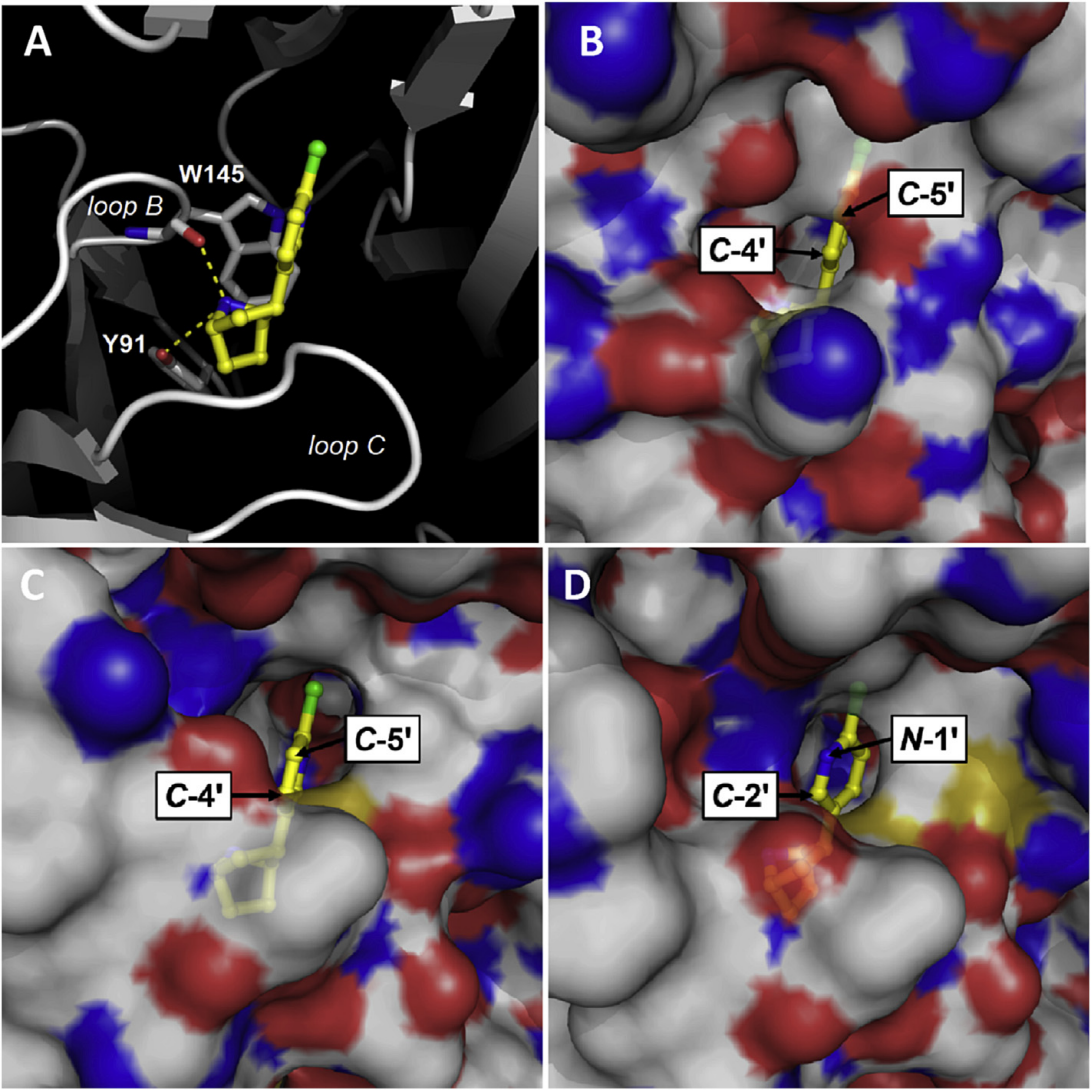
A comparison of the orientations of epibatidine (yellow, displayed as a ball-and-stick model) in the different binding sites of 2BYQ and 3SQ6, showing the accessibility of different regions of the ligand. **A, B**. The surface of the protein has been hidden in panel A to show the location of epibatidine in the binding site, relative to binding loops B and C. The orientation shown in panel B is the same as that in panel A, but with the protein surface visible. This is the only orientation found in all ten binding sites from 3SQ6 and the C*-*4′ position is most accessible. **C**. Accessibility of the C*-*5′ position is seen in 2BYQ where it is found in four of the five binding sites. **D**. In 2BYQ, one of the binding poses (chains B & A) also exposes the *N-*1′ position. The atom numbering used in this figure can be seen in Fig. 1. Potential hydrogen bonds are indicated by yellow dashes. (For interpretation of the references to colour in this figure legend, the reader is referred to the web version of this article.)

In the principal binding interface of the α7 nACh receptor, Cys substitution of Y91 in loop A abolished binding, indicating a key interaction. In the AChBP co-crystal structure 3SQ6, Y91 forms a cation-π interaction with K141 and a hydrogen bond with epibatidine (Fig. 5A), both of which have also been observed in functional assays on α7 nACh receptors. For example, removing the hydrogen bonding capability of Y91 by substitution with Phe caused a dramatic reduction in the affinity of α7 nACh for epibatidine, while unnatural amino acid mutagenesis identified a cation-π interaction at the same location (Li et al., 2011, Puskar et al., 2011). In loop B, Cys substitution of the aromatic residue W145 also abolished epibatidine binding, indicating another important effect. This centrally located aromatic residue is highly conserved across the Cys-loop family and even conservative substitutions at this location can impact ligand binding (Li et al., 2011, Puskar et al., 2011, Thompson et al., 2010, Van Arnam et al., 2013, Williams et al., 2009). In both of the AChBP crystal structures this residue is seen to form a cation-π interaction with epibatidine, but unnatural amino acid mutagenesis of α7 nACh receptors suggests that such an interaction is not present (Puskar et al., 2011). For other ligands an interaction at this location is not always essential for agonist function at α7 nACh receptors, as shown by mutations to Ala and Gly that cause only small changes in the function of ACh and no change for the compound 4OH-GTS-2 (Williams et al., 2009). In contrast, our results suggest that W145 has a more important role for epibatidine, and are consistent with findings that even a conservative W145F mutation at α7 nACh receptors reduces the affinity of epibatidine by > 2200-fold (Li et al., 2011). At other residue positions in Loop B the effects of mutation were not as pronounced. For example, S144 and T146 lay either side of W145, and although mutation of these residues can have quite dramatic effects for ligands acting at other Cys-loop receptors, it had little or no effect on epibatidine (Thompson et al., 2010). Based on crystal structure evidence, both of these residues contribute to a network of hydrogen bonds, and it is therefore possible that the ability of the thiol side chain of Cys (which can also act as a weak hydrogen bond donor) might preserve this property. Elsewhere mutation of other loop B residues K141 and G149 had only minor effects on the *EC*_50_ or the binding affinity of epibatidine at α7 nACh receptors and as both are located distant (>6.5 Å) from the bound ligand in AChBP our results are also consistent with these previous findings (Criado et al., 2005, Grutter et al., 2003). In loop C, no epibatidine binding was detected following the substitution Y191C. Consistent with this result, mutation of Y191 to Phe decreases its affinity and unnatural amino acid mutagenesis has provided evidence that Y191 forms a cation-π interaction with epibatidine at α7 nACh receptors (Li et al., 2011, Puskar et al., 2011). In AChBP the closely located residues Y184, C186 and C187 make extensive van der Waals contacts with epibatidine via its azabicyclo moiety (Hansen et al., 2005, Li et al., 2011). In our study of α7 nACh receptors, epibatidine binding was not detectable following Y184C substitution, a finding that is comparable to the >250-fold change that was previously reported following the modest substitution Y184F (Li et al., 2011). We also found that Ala mutation of either of the closely located vicinal disulphides (C186, C187) decreased the affinity for epibatidine by > 30-fold. This is consistent with the increased *EC*_50_ of ACh that is seen when the equivalent residue is mutated in other nACh receptor subtypes such as α1 subunit-containing nACh receptors (Blum et al., 2011, Karlin and Bartels, 1966). In α1 nicotinic subunits the disulphide bond formed by these adjacent Cys is proposed to distort loop C and enable key residues to interact with loop F on the opposite side of the binding interface, and at α7 nACh receptors they may have a similar role (Blum et al., 2011).

On the complementary face of the binding site, W53 in loop D also constitutes part of the aromatic box. We show that binding is undetectable following Cys mutation of this residue at α7 nACh receptors. In the AChBP crystal structure 3SQ6, W53 stabilises W145 via edge-to-face interactions rather than making a direct contact with epibatidine. Such an interaction is consistent with the relatively small affinity change (4-fold) for epibatidine when a conservative aromatic W53F substitution is made and the larger reduction (17-fold; from 0.34 μM to 5.8 μM) in the *EC*_50_ of epibatidine when the same residue is substituted by Ala (Li et al., 2011, Van Arnam et al., 2013). In *Aplysia* AChBP (2BYQ) the same position is a Tyr, and mutating this to Trp increase the affinity of epibatidine to a value more closely resembling α7 nACh receptors, indicating that stabilisation of W145 may be more optimal with a larger aromatic residue at position 53 (Hansen et al., 2005). Q55C is closely located to W53, but the affinity of epibatidine at the mutant receptor was unaltered when compared to wild type α7 nACh receptors. A similar absence of major effects following Cys substitution of this residue have been reported in functional studies of other agonists at α7 nACh receptors (Papke et al., 2011). Based on these findings and the orientation of epibatidine in both of the co-crystal structures it is unlikely that this residue makes substantial contact with the ligand or affects gating. Also present in the complementary binding interface, the AChBP crystal structures indicate that the equivalent of loop E residues N104, L106, Q114 and L116 stabilise epibatidine via Van der Waals interactions with its chloropyridine ring (Hansen et al., 2005, Li et al., 2011). We found that the binding affinity was unaltered by Cys substitution of L106 and L116, possibly owing to the similar size and hydrophobicity of leucine and Cys (Nagano et al., 1999). For L106 our results are the same as those for L106A which does not affect the *EC*_50_ of epibatidine, ACh or varenicline (Van Arnam et al., 2013). In contrast, our results for L116 appear to conflict with reports that showed Ala mutation causes a 9-fold change in the *EC*_50_ of epibatidine, and that the equivalent residue makes an important hydrogen-bond with agonists in α4β2 nACh receptors (Blum et al., 2013, Van Arnam et al., 2013). However, when the backbone of L116 was probed by unnatural amino acid mutagenesis to determine if hydrogen-bonding was important at α7 nACh receptors, there were only minor effects on the binding of epibatidine, ACh and varenicline, suggesting that this residue does not have the same role as that found in α4β2 nACh receptors (Van Arnam et al., 2013). Furthermore, a lack of effects on activation by other agonists has been previously seen following Cys substitution at L116, and changes may be dependent upon the ligand and the residue substitution (Amiri et al., 2008, Papke et al., 2011). For Q114 a halogen-bonding interaction between the backbone carbonyl and the pyridine chlorine of epibatidine is seen in AChBP (Li et al., 2011). Consistent with this, removal of the side chain of Q114 by substitution to Ala has little effect on the *EC*_50_ of epibatidine and other α7 nACh receptor agonists (Van Arnam et al., 2013). We did not probe main chain interactions, but our findings suggest that Cys mutation may have additional effects. For example, as Q114C increased the affinity for epibatidine by 5-fold, we speculate that this could reflect the formation of an attractive non-covalent interaction between the sulfhydryl group and the chlorine atom. Elsewhere in AChBP mutation of residues in loop C is similarly reported to increase the affinity of ACh and it was also speculated that this might reflect changes in electrostatic interactions (Li et al., 2011). In loop F, neither D160C nor S162C mutations had any effect on the affinity of epibatidine, nor indeed are there any previous reports of these residues mediating ligand interactions or functional effects. In other studies, mutation of the closely located residue G167 also had no effect on the function of epibatidine when mutated to a range of other amino acids, and I161C and G167C had no effect on several other α7 nACh receptor agonists, further indicating that this region may not have a major role in binding (Matsuda et al., 2000, Papke et al., 2011).

High-affinity epibatidine binding has also been reported at 5-HT_3_ receptors, similar to the binding of ACh, nicotine, tropisetron and *d*-tubocurarine at the two receptor types (ChavezNoriega et al., 1997, Drisdel et al., 2008, Thompson et al., 2014). In light of this we also measured binding at the 5-HT_3_ receptor to establish whether epibatidine could have utility for studying this other member of the Cys-loop receptor family. However, saturable binding with [^3^H]epibatidine was not detected at either human or mouse 5-HT_3_ receptors at a concentration of up to 25 nM. This result was confirmed when competition of unlabelled epibatidine with the 5-HT_3_ receptor antagonist [^3^H]tropisetron yielded a p*K*_i_ of 5.60 ± 0.02 (*K*_i_ = 2.51 μM, *n* = 3) for human and a p*K*_i_ of 4.91 ± 0.12 (*K*_i_ = 12.3 μM, *n* = 4) for mouse, but contrasts with a report from Drisdel et al. (2008) who found a nanomolar affinity for epibatidine at 5-HT_3_ receptors. It is unclear why there is a 100-fold difference between our results, but the larger value we measured precludes the possibility of using this ligand as precursor for developing novel molecular probes for studying 5-HT_3_ receptors. In contrast, epibatidine may provide an opportunity for developing α7 nACh receptor probes, but previous efforts to couple fluorophores at the C-5′ position (see Fig. 1 for atom numbering) caused a dramatic reduction in affinity (at worst, from nM to mM) and made the resultant fluorescent derivatives unsuitable for fluorescence-based experiments (Grandl et al., 2007). However, further analysis of 3SQ6 suggests that the C-4′ position is more accessible than the C-5′ position. Indeed, all ten of the binding orientations in 3SQ6 (Fig. 5B) expose the C-4′ position, while orientations in 2BYQ show that either the C-5′ is more accessible or that the pyridine ring is flipped by 180° which makes the C-2′ most accessible (Fig. 5C and D). The finding that C-5′ substituted epibatidine derivatives still bind within the tightly confined space of the ligand binding site shows that conjugation at this location does not prevent binding, but the modification is not optimal. Our finding that the orientations of epibatidine in the AChBP co-crystal structures are broadly correct, and that 3SQ6 shows improved accessibility at the C-4′ position, suggests that there may still be opportunities to develop epibatidine-based probes with higher affinities for the α7 nACh receptor. In our previous work the development of similar probes for targeting 5-HT_3_ receptors has enabled quantitative fluorescent measurements, receptor localisation and photo cross-linking to be performed, and shows the value of pursing their synthesis (Lochner and Thompson, 2015, Jack et al., 2014).

To visualise ligand-receptor interactions homologous proteins are often used as substitutes for the native receptor, and AChBP is a popular substitute for all Cys-loop receptors despite often quite distinct pharmacologies for the same ligands at different receptors. Here we specifically examined another of the structures, to determine whether changes in the affinity of epibatidine caused by point mutations in the α7 nACh receptor are consistent with residues identified in epibatidine-bound AChBP crystals. The results presented here suggest that the orientations of epibatidine in the AChBP crystal structures are a reasonable approximation of its orientation at α7 nACh receptors. It is also striking how similarly epibatidine binds in the two crystal structures despite the only 31.8% sequence identity between 2BYQ and 3SQ6. This similarity in ligand orientation extends to the *in silico* docking presented here and perhaps reflects the high conservation of major aromatic residues that constitute the binding site (in 3SQ6 and the α7 nACh receptor these residues are identical) and the tight pocket imposed by the agonist-bound conformation. Furthermore, while the accessibility of locations on the pyridine ring of epibatidine still needs to be clarified in more detail, we can now be more confident of the crystal structures and use them as models for our ongoing efforts to attach fluorescent moieties to this ligand in order to develop specific high-affinity molecular probes for studying the α7 nACh receptor subtype. The use of cysteine mutants could also provide a stimulus for probing ligand interactions by chemical modification (e.g. by methanethiosulfonate (MTS) reagents) of the thiol side chains.

## Figures and Tables

**Fig. 4:**
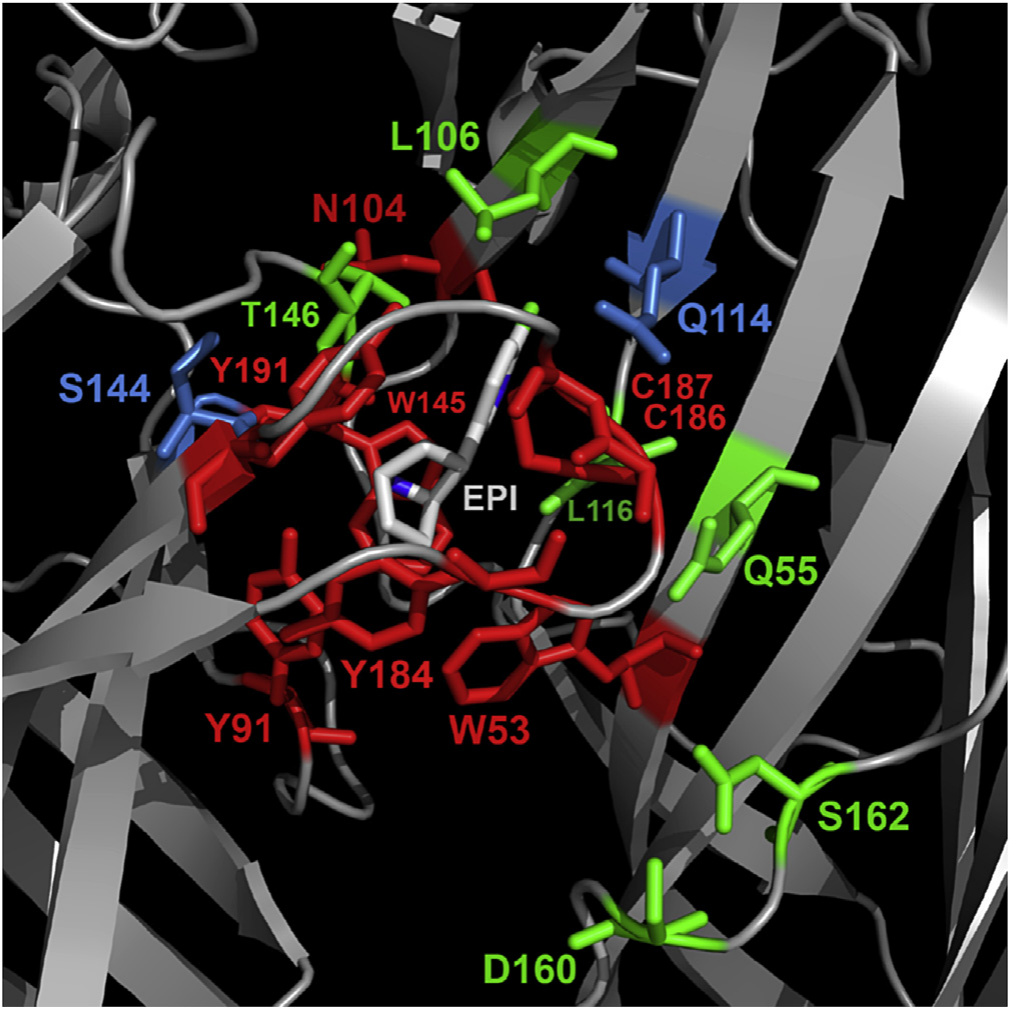
Binding site residues mutated in this study, highlighted on the AChBP receptor crystal structure 3SQ6 and colour coded by whether substitution caused no change in the affinity for epibatidine (green), decreased affinity (red), or increased affinity (blue). The affinities of each mutant and the fold-change relative to the wild-type receptor can be found in Table 2. EPI = epibatidine. (For interpretation of the references to colour in this figure legend, the reader is referred to the web version of this article.)

**Table 1:** Summary of all residues lying within 5 Å of epibatidine.

	Principal Face									Complementary Face										
PDB id	A		B			C					D		E							F
**3SQ6**	Y91		S144	W145	T146	Y184	C186	C187	Y191		W53		L104	A105	L106	Q114	Y115	L116		
**2BYQ**	Y	T	S	W	T	Y	C	C	Y		Y		I	A	V	M	F	I		
**1UV6**	Y		S	W	T	Y	C	C	Y		W		L	A	R	L	Y	M		
**1UW6**	Y		S	W	T	Y	C	C	Y		W		L	A	R	L	Y	M		
**3WIP**	Y		S	W	T	Y	C	C	Y		W		L	A	R	L	Y	M		
**4AFT**	Y		S	W	V	Y	C	C	Y		Y	R	I	A	V	M	F	I		
**2WNC**	Y		S	W	V	Y	C	C	Y	Q	Y		I	A	V	M	F	I		S
**2WNL**	Y		S	W	V	Y	C	C	Y		Y		I	A	V	M	F	I		
**4BQT**	Y		S	W	V	Y	C	C	Y		Y	R	I	A	V	M	F	I	P	

Locations of the most predominant residues can be found in Fig. 1, Fig. 4. Only the numbering for 3SQ6 has been included to avoid confusion. 3SQ6 and 2BYQ are co-crystal structures of epibatidine bound to AChBP. Epibatidine has been docked into the remaining structures shown. A – F = binding loops (see Fig. 1).

**Table 2:**
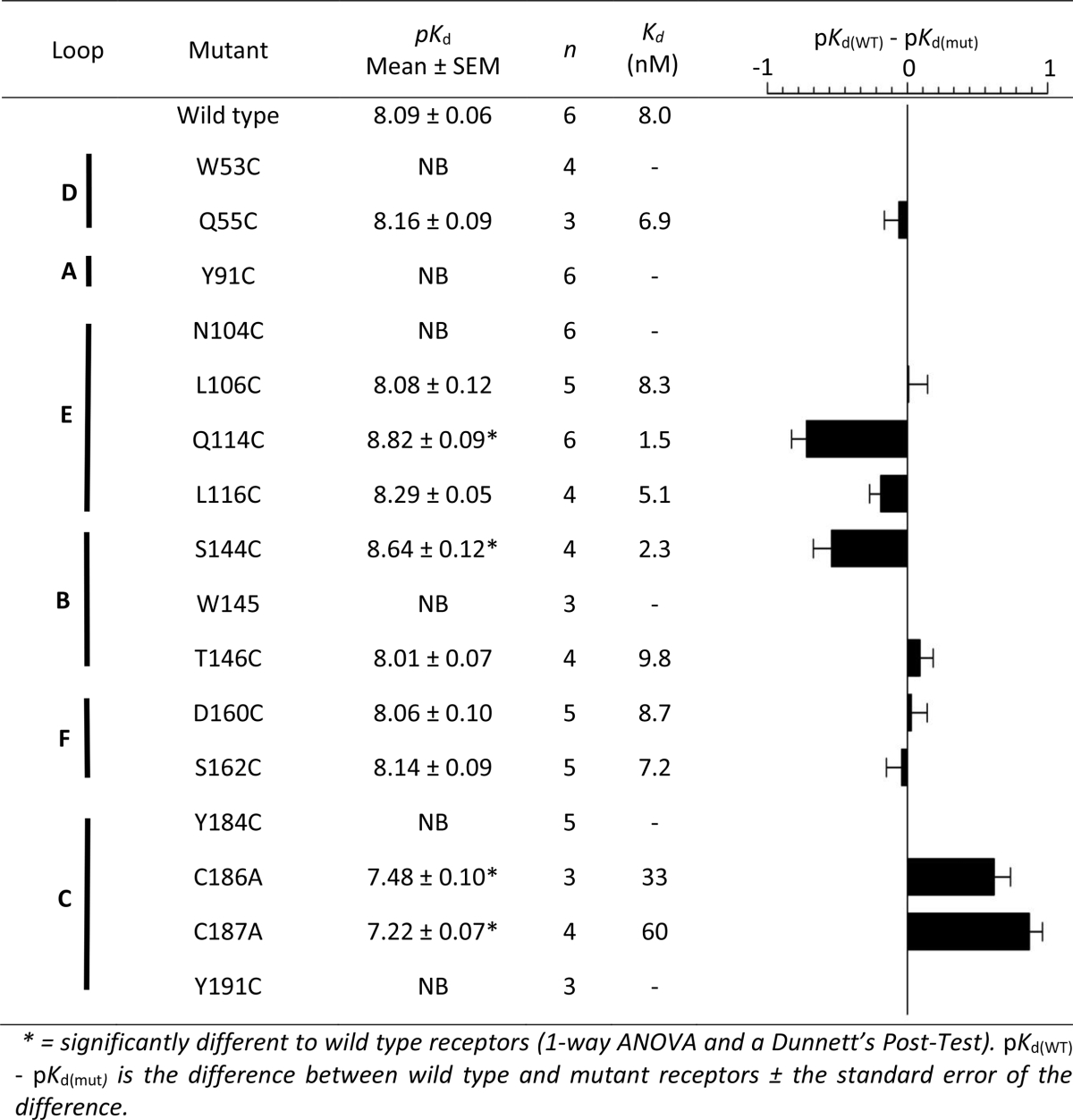
Saturation binding of [^3^H]epibatidine at mutant α7 nACh receptors.

## References

[bib1] Akdemir A., Edink E., Thompson A.J., Lummis S.C., Kooistra A.J., de Graaf C. (2012). Identification of novel alpha7 nicotinic receptor ligands by *in silico* screening against the crystal structure of a chimeric α7 receptor ligand binding domain. Bioorg. Med. Chem..

[bib2] Amiri S., Shimomura M., Vijayan R., Nishiwaki H., Akamatsu M., Matsuda K. (2008). A role for Leu118 of loop E in agonist binding to the α7 nicotinic acetylcholine receptor. Mol. Pharmacol..

[bib3] Blum A.P., Gleitsman K.R., Lester H.A., Dougherty D.A. (2011). Evidence for an extended hydrogen bond network in the binding site of the nicotinic receptor. Role of the vicinal disulphide of the α 1 subunit. JBC.

[bib4] Blum A.P., Van Arnam E.B., German L.A., Lester H.A., Dougherty D.A. (2013). Binding interactions with the complementary subunit of nicotinic receptors. JBC.

[bib5] Camacho-Bustamante G.A., Kaczanowska K., Harel M., Cornejo-Bravo J., Taylor P. (2016). FASEB J..

[bib6] ChavezNoriega L.E., Crona J.H., Washburn M.S., Urrutia A., Elliott K.J., Johnson E.C. (1997). Pharmacological characterization of recombinant human neuronal nicotinic acetylcholine receptors h α2β2, h α2β4, h α3β2, h α3β4, h α4β2, h α4β4 and h α7 expressed in *Xenopus* oocytes. JPET.

[bib7] Colquhoun D. (1998). Binding, gating, affinity and efficacy: the interpretation of structure-activity relationships for agonists and of the effects of mutating receptors. BJP.

[bib8] Criado M., Mulet J., Bernal J.A., Gerber S., Sala S., Sala F. (2005). Mutations of a conserved lysine residue in the N-terminal domain of α7 nicotinic receptors affect Gating and binding of nicotinic agonists. Mol. Pharmacol..

[bib9] Drisdel R.C., Sharp D., Henderson T., Hales T.G., Green W.N. (2008). High affinity binding of epibatidine to serotonin type 3 receptors. JBC.

[bib10] Eiselé J.-L., Bertrand S., Galzi J.-L., Devillers-Thiéry A., Changeux J.-P., Bertrand D. (1993). Chimaeric nicotinic-serotonergic receptor combines distinct ligand binding and channel specificities. Nature.

[bib11] Grandl J., Sakr E., Kotzyba-Hibert F., Krieger F., Bertrand S., Bertrand D. (2007). Fluorescent epibatidine agonists for neuronal and muscle-type nicotinic acetylcholine receptors. Angew. Chem. Int. Ed..

[bib12] Grutter T., Prado de Carvalho L., Le Novere N., Corringer P.J., Edelstein S., Changeux J.P. (2003). An H-bond between two residues from different loops of the acetylcholine binding site contributes to the activation mechanism of nicotinic receptors. EMBO J..

[bib13] Hansen S.B., Sulzenbacher G., Huxford T., Marchot P., Taylor P., Bourne Y. (2005). Structures of Aplysia AChBP complexes with nicotinic agonists and antagonists reveal distinctive binding interfaces and conformations. EMBO J..

[bib14] Hernandez C.M., Dineley K.T. (2012). α7 nicotinic acetylcholine receptors in Alzheimer's disease: neuroprotective, neurotrophic or both?. Curr. Drug Targets.

[bib15] Jack T., Ruepp M.-D., Thompson A.J., Mühlemann O., Lochner M. (2014). Synthesis and characterization of photoaffinity probes that target the 5-HT_3_ receptor. Chimia.

[bib16] Karlin A., Bartels E. (1966). Effects of blocking sulfhydryl groups and of reducing disulfide bonds on acetylcholine-activated permeability system of electroplax. Biochim. Biophys. Acta.

[bib17] Li S.X., Huang S., Bren N., Noridomi K., Dellisanti C.D., Sine S.M. (2011). Ligand-binding domain of an α7-nicotinic receptor chimera and its complex with agonist. Nat. Neurosci..

[bib18] Lochner M., Thompson A.J. (2015). A review of fluorescent ligands for studying 5-HT_3_ receptors. Neuropharmacol.

[bib19] Lummis S.C., Thompson A.J., Bencherif M., Lester H.A. (2011). Varenicline is a potent agonist of the human 5-hydroxytryptamine_3_ receptor. JPET.

[bib20] Macor J.E., Gurley D., Lanthorn T., Loch J., Mack R.A., Mullen G. (2001). The 5-HT_3_ antagonist tropisetron (ICS 205-930) is a potent and selective α7 nicotinic receptor partial agonist. Bioorg. Med. Chem. Lett..

[bib21] Matsuda K., Shimomura M., Kondo Y., Ihara M., Hashigami K., Yoshida N. (2000). Role of loop D of the α7 nicotinic acetylcholine receptor in its interaction with the insecticide imidacloprid and related neonicotinoids. BJP.

[bib22] Mazurov A.A., Speake J.D., Yohannes D. (2011). Discovery and development of α7 nicotinic acetylcholine receptor modulators. J. Med. Chem..

[bib23] Nagano N., Ota M., Nishikawa K. (1999). Strong hydrophobic nature of Cys residues in proteins. Febs Lett..

[bib24] Papke R.L., Stokes C., Williams D.K., Wang J., Horenstein N.A. (2011). Cys accessibility analysis of the human α7 nicotinic acetylcholine receptor ligand-binding domain identifies L119 as a gatekeeper. Neuropharmacol.

[bib25] Puskar N.L., Xiu X., Lester H.A., Dougherty D.A. (2011). Two neuronal nicotinic acetylcholine receptors, α4β4 and α7, show differential agonist binding modes. JBC.

[bib26] Rice P., Longden I., Bleasby A. (2000). EMBOSS: the European molecular biology open software suite. Trends Genet..

[bib27] Thompson A.J., Lester H.A., Lummis S.C. (2010). The structural basis of function in Cys-loop receptors. Q. Rev. Biophys..

[bib28] Thompson A.J., Verheij M.H.P., Verbeek J., Windhorst A.D., de Esch I.J.P., Lummis S.C.R. (2014). The binding characteristics and orientation of a novel radioligand with distinct properties at 5-HT_3_A and 5-HT_3_AB receptors. Neuropharmacol.

[bib29] Thomsen M.S., Hansen H.H., Timmerman D.B., Mikkelsen J.D. (2010). Cognitive improvement by activation of α7 nicotinic acetylcholine receptors: from animal models to human pathophysiology. Curr. Pharm. Des..

[bib30] Unwin N. (2005). Refined structure of the nicotinic acetylcholine receptor at 4Å resolution. J. Mol. Biol..

[bib31] Van Arnam E.B., Blythe E.E., Lester H.A., Dougherty D.A. (2013). An unusual pattern of ligand-receptor interactions for the α7 nicotinic acetylcholine receptor, with implications for the binding of varenicline. Mol. Pharmacol..

[bib32] Williams D.K., Stokes C., Horenstein N.A., Papke R.L. (2009). Differential regulation of receptor activation and agonist selectivity by highly conserved Trps in the nicotinic acetylcholine receptor binding site. JPET.

[bib33] Xiao Y.D., Hammond P.S., Mazurov A.A., Yohannes D. (2012). Multiple interaction regions in the orthosteric ligand binding domain of the α7 neuronal nicotinic acetylcholine receptor. J. Chem. Inf. Mod..

[bib34] Yamauchi J.G., Gomez K., Grimster N., Dufouil M., Nemecz A., Fotsing J.R. (2012). Synthesis of selective agonists for the α7 nicotinic acetylcholine receptor with *in situ* click-chemistry on acetylcholine-binding protein templates. Mol. Pharmacol..

[bib35] Yang Z., Ney A., Cromer B.A., Ng H.L., Parker M.W., Lynch J.W. (2007). Tropisetron modulation of the glycine receptor: femtomolar potentiation and a molecular determinant of inhibition. J. Neurochem..

